# Endo-cost: efficient economic model of adopting robotic versus laparoscopic gynecological surgery for endometrial cancer

**DOI:** 10.1007/s11701-025-02706-6

**Published:** 2025-11-03

**Authors:** Sara García-Álvarez, Marta Arnáez De la Cruz, Iria Rey, Pablo Padilla-Iserte, Luis Matute, Marta Gurrea, Santiago Domingo, María Caballer, Victor Lago

**Affiliations:** 1 Economic Policy and Public Economics PhD School, University of Valencia, Valencia, Spain; 2 Department of Gynecologic Oncology, Hospital Universitari i Politecnic La Fe, Valencia, Spain; 3 Gynecological Tumours, Breast and Hereditary Cancer Research Group, La Fe Health Research Institute (IISLAFE), Avinguda de Fernando Abril Martorell, 106, 46026 Valencia, Spain; 4 Department of Obstetrics and Gynecology, University of Valencia, Valencia, Spain; 5 Department of Applied Economics, University of Valencia, Valencia, Spain; 6 CEU Cardenal Herrera University, Valencia, Spain

**Keywords:** Endometrial cancer, Laparoscopy, Robotic surgery, Economic evaluation

## Abstract

This retrospective cost-analysis study evaluated and compared the economic and clinical outcomes of laparoscopic versus robotic surgery in the treatment of endometrial cancer at the University and Polytechnic La Fe Hospital in Valencia, Spain. The analysis included 153 patients who underwent surgery between January 2022 and December 2024. Robotic surgery resulted in a shorter average hospital stay (1.12 vs. 1.7 days; *p* < 0.05) compared to laparoscopy, but was associated with significantly higher costs in both consumables (€1985 vs. €1197; *p* < 0.001) and fixed amortization (€2000 vs. €140; *p* < 0.001) and total cost per procedure (€4698 vs. €7052 ± 816; *p* < 0.001). No significant differences were observed in surgical time or postoperative complications. A theoretical cost model based on the high procedural volume (three robotic surgeries per day, 7920 total over ten years) still showed laparoscopy to be more cost-effective overall (€4596 vs. €5431 per procedure; *p* < 0.001). However, robotic surgery could become economically competitive with a 15% reduction in equipment costs and a 30% decrease in consumable expenses, provided at least two procedures are performed daily. Ultimately, although laparoscopy remains more cost-efficient under typical conditions, robotic surgery offers potential economic viability under optimized circumstances, supporting its consideration as a sustainable option in high-volume centers managing endometrial cancer.

**Supplementary Information:**

The online version contains supplementary material available at 10.1007/s11701-025-02706-6.

## Introduction

Robotic-assisted surgeries emerged in the 1990s, and over time, several robotic systems have been developed (Eg. Da Vinci®, HUGO®, Versius®). Today, the Da Vinci® system is the most widely used in different types of surgery, including gynecological surgery [1]. However, the high cost of acquisition and maintenance limits its use, and debate persists as to whether its benefits justify the associated expense [2]. The main handicap to its implementation is that an efficient and effective surgical technique, such as laparoscopy is available. In endometrial cancer, laparoscopy is the preferred option in early stages, demonstrating no inferiority in terms of survival and disease control, along with advantages in postoperative recovery when compared with laparotomy [3, 4].

In Europe, its implementation and expansion face budgetary constraints and clinical effectiveness evaluations [5]. European economic studies comparing laparoscopic (LPS) and robotic surgery (RBT) for endometrial cancer treatment [6–12] show some limitations of the advantages of robotic surgery: both presented similar average operative times (214 vs. 210 min) but lower intraoperative complication rates in robotic surgery (6.2% vs. 2.1%). The transfusion rate is similar in both groups (15.5% vs. 14.3%) while the mean hospital stay is longer in case of laparoscopic surgery (4.7 vs. 3.2 days), which might be its principal advantage. The range of increased costs per procedure for robotic surgery compared to laparoscopy is between 8 and 38% [8, 12]. For the exposed reasons, there are concerns about the economic viability of robotic surgery representing a barrier to its implementation. Therefore, we propose a study to stablish the bases for an economic model to facilitate and guide health economic investment policies of robotic surgery in our environment.

## Materials and methods

We have designed a retrospective comparative study to evaluate the costs of robotic and laparoscopic surgery for endometrial cancer. The study was approved by the hospital’s ethics committee on 30 July 2024 (Local cod 2024-57). The medical records of patients who underwent surgery for endometrial cancer between January 2022 and December 2024 at the University and Polytechnic La Fe Hospital in Valencia were reviewed. The learning curve had been fully overcome by all surgeons prior to the inclusion period.

### Objectives

The primary objective was to establish and compare the cost per procedure of laparoscopic vs robotic surgery in the surgical treatment of endometrial cancer in our environment (tertiary reference center). The secondary objectives were to compare both the clinical and economic variables between laparoscopic and robotic surgery in endometrial cancer; to establish the number of cost-effective procedures (laparoscopic surgery vs. robotic surgery) and to establish the cost-effectiveness threshold of robotic surgery from a theoretical framework.

### Selection criteria

The inclusion criteria were as follows: Patients with apparently early-stage endometrial cancer (FIGO I-II), negative CT scan or MRI for distant disease and surgery performed by minimally invasive means. The exclusion criteria were: Laparotomic or vaginal surgery, indication for surgery for other gynecological causes (benign pathology or gynecological cancer other than endometrial cancer e.g. cervical, ovarian).

### Clinical variables

We collected information on the following clinical variables: age, medical conditions, body mass index, previous surgeries, parity, ASA scale (anesthetic risk scale), type of surgical procedures, surgical consumables used in surgery, duration of surgery, complications during surgery, estimated blood loss, need for intraoperative transfusion, days of hospitalization, postoperative complications according to the Clavien–Dindo classification [13]. Surgical field preparation was defined as the time elapsed between the first incision and the initiation of the scheduled procedure, including tasks such as organ pexy, adhesiolysis, bowel mobilization, docking, among others. The type of surgery was classified according to increasing complexity and was categorized as follows: Group A: Hysterectomy + Double adnexectomy; Group B: Hysterectomy + Double adnexectomy + Sentinel lympnode; Group C: Group A or B + Lymphadenectomy ± Omentectomy. For the cost estimation, we obtained and analyzed all the resources consumed in each different surgical procedure, postoperative period and complications derived from the intervention.

### Economic variables

In order to obtain information on direct costs (consumables and inventories) and indirect costs, we have used public sources of information: public bidding documents from the Spanish Ministry of Health: Plataforma de Contratación del Sector Público, file number 224/2022; the economic department of La Fe Hospital in Valencia: supply section and section of economic information and complementary services of the Valencia La Fe Health Department and information available from medical devices companies: Storz and Abex.

Costs were divided into direct and indirect, all calculated pre-tax. Indirect costs included operating theatre use and hospital stay. The theatre cost was €14.65 per minute, covering facility use, medical staff (2 surgeons, 3 nurses and 1 anesthesiologists, identical for both type of procedure), and medications used during surgery. Hospital stay cost €681 per day, including room, meals, postoperative care, medical staff, and materials used during recovery. All included procedures were performed after the learning curve had been surpassed; therefore, staff training costs were not taken into consideration.

Direct costs included inventoriable material used in surgery (equipment) and Surgical consumables used in each surgery. Inventoriable material used refers to equipment and machinery that can be used repeatedly in successive procedures until the end of its useful life. This material differs in laparoscopic and robotic surgery. Regarding the equipment needed in laparoscopy, we refer to the minimum elements required to constitute a laparoscopic ‘tower’, which include: 2 monitors, light source, light cable, indocyanine green (ICG) system, camera unit, optics, CO2 insufflator, high frequency unit and power console. The price in our center was €140,000 according to information available from reference medical device company (Storz®). Regarding the equipment necessary for robotic surgery, we refer to the minimum essential elements that consist of: Patient trolley (Da Vinci Xi®), operating table, viewing tower (intercom, touch screen, electrosurgical console, camera unit), optics, AirSeal® console and surgical console. Its price in our center was €2,000,000 according to public bidding documents from the Spanish government. Surgical consumables used in surgery refers to material that is used once per surgery (cost of a single use) or material that has a pre-established number of uses (its cost has been calculated for one use), as in the case of robotic instruments, given that some of the instruments have between 10 and 18 uses. Maintenance of the robotic platform was included as part of the direct costs.

### Statistical analysis

An initial exploratory analysis was conducted to assess data quality and identify missing values. Statistical significance was set at *p* < 0.05. Qualitative variables were analyzed using the Chi-squared test, or Fisher’s test when expected frequencies were below five. For quantitative variables, normality was tested with the Kolmogorov–Smirnov test. If normally distributed, comparisons used the Student’s *t* test; otherwise, the Wilcoxon test was applied. A multivariate analysis using binary logistic regression examined the relationship between surgery type and predictor variables. A theoretical ratio was then developed to estimate the number of cost-effective procedures for each technique. Finally, a cost minimization analysis was conducted by modeling different scenarios for robotic surgery, varying direct costs and procedure volume. All analyses were performed using R Studio, version 4.4.1.

## Results

A total of 153 records were included in the analysis. The results in Table 1 show a comparison of clinical, surgical and postoperative variables between the groups of patients undergoing LPS (*n* = 75) and RBT (*n* = 78) surgery. No significant differences were found in baseline clinical variables in terms of age, BMI, or history of previous surgery. Regarding surgical variables, the groups presented similar distributions in terms of surgical complexity score and conversion rate to laparotomy. Previous preparation of the surgical field was significantly more frequent in the robotic group with 16.7% versus 1.3% in the laparoscopic group (*p* = 0.001). There were no differences regarding the types of procedures performed. There were no statistical differences regarding duration of surgery between groups (161 vs 152 min; *p* = 0.556). Intraoperative and postoperative complications were infrequent, making statistical comparison between groups potentially unreliable. Regarding postoperative complications, in the laparoscopic group there was a suspected ureteric thermal injury during the procedure, which was managed with prophylactic JJ catheter insertion and resolved without sequelae. In the robotic group, two minor vascular injuries occurred during SLN dissection: both were venous injuries successfully managed with simple sutures, without transfusion requirements or further consequences.

**Table 1:** Univariate analysis of clinical, surgical, and postoperative variables

	LPS (*n*75)	RBT (*n*78)	*P* value
Age, years; mean (SD)	65.1 (10.5)	65.4 (11.9)	0.8954
BMI, kg/m^2^; mean (SD)	29.1 (5.6)	30.5 (7.4)	0.3235
BMI classification; *n* (%)			0.2169
Normal weight	22 (29.3)	24 (30.8)	
Overweight	23 (30.7)	15 (19.2)	
Obesity	26 (34.7)	29 (37.2)	
Morbid obesity	4 (5.3)	10 (12.8)	
Previous surgery; *n* (%)	26 (34.7)	29 (37.2)	0.7461
Surgical complexity score; *n* (%)			0.7833
A	14 (18.7)	13 (16.7)	
B	46 (61.3)	52 (66.7)	
C	15 (20)	13 (16.7)	
Surgical field preparation; *n *(%)	1 (1.3)	13 (16.7)	<0.001*
Conversion to laparotomy; *n* (%)	1 (1.3)	0 (0)	0.4902
Surgical procedures; *n* (%)			
SLNB	56 (74.7)	68 (87.2)	0.048
Pelvic lymphadenectomy	10 (13.3)	5 (6.4)	0.15
Para-aortic lymphadenectomy	1 (1.3)	0 (0)	0.4902
Omentectomy	9 (12)	8 (10.3)	0.7316
Urological complication	1 (1.3)	0 (0)	0.4902
Vascular complication	0 (0)	2 (2.6)	0.4969
Surgery duration, min; mean (SD)	161 (60)	152 (43)	0.5562
Postoperative complications; *n* (%)			
Clavien–Dindo Grade I	1 (1.3)	1 (1.3)	1
Clavien–Dindo Grade II	0 (0)	0 (0)	–
Clavien–Dindo Grade III	2 (2.7)	0 (0)	0.2386
Hospital stay, days; mean (SD)	1.7 (1.02)	1.12 (0.46)	<0.001*

LPS: Laparoscopic; RBT: Robotic; BMI: Body mass index; SLNB: Sentinel lymph node biopsy

Regarding postoperative complications classified according to the Clavien–Dindo system, there were no significant differences between groups: one grade I complication in each group, and two grade III complications in the laparoscopic group (resuturing of the vaginal cuff and drainage of an abdominal collection). The mean length of hospital stay was significantly longer in the laparoscopic group at (mean ± SD) 1.7 ± 1.02 days compared to 1.12 ± 0.46 days, in the robotic group, being this difference statistically significant (*p* < 0.001).

**Fig. 1:**
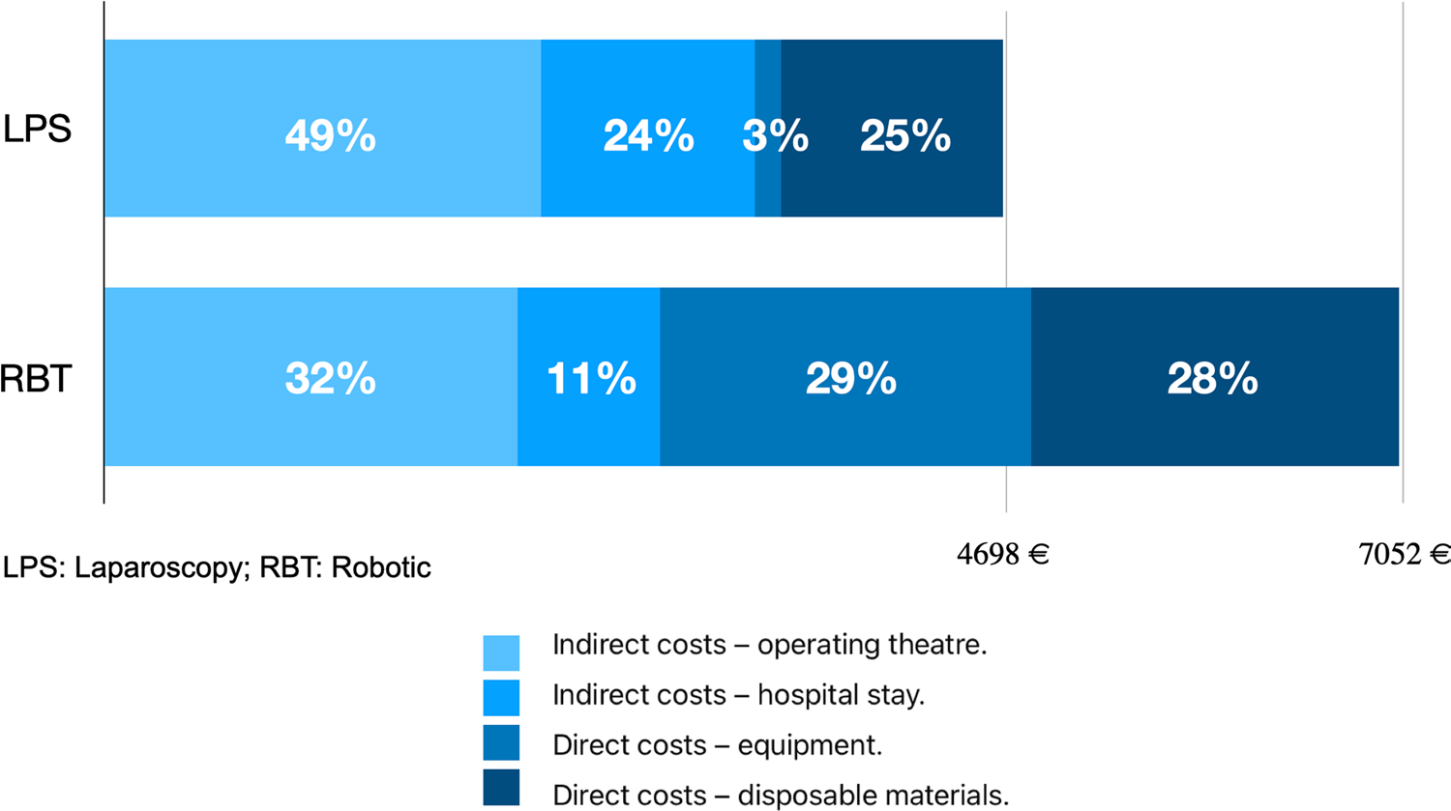
Percentage of total cost per procedure

Table 2 presents a univariate analysis of economic variables comparing the 2 groups, analyzing indirect and direct costs. There were no differences regarding the indirect operating room costs between the two groups (€2356 vs. €2227; *p* = 0.556), while direct costs (consumables: €1047 vs. €2057; *p* < 0.001; and equipment: €140 vs. €2000; *p* < 0.001) and indirect costs of stay were significantly higher in the robotic procedures (€1153 vs. €768; *p* < 0.001). The total cost per procedure, which includes all the aforementioned cost, was also significantly higher in the robotic group (€4698 ± 1121 vs. €7052 ± 816; *p* < 0.001). Figure 1 shows the percentage that each category represents of the total cost.

**Table 2:** Univariate analysis of economic variables of interest

Variable	LPS (*n*75)	RBT (*n*78)	*P* value
Indirect costs (operating room), €; mean (SD)	2356 (880)	2227 (627)	0.556
*The calculation was made by multiplying the duration of operating room use in minutes (recorded in the surgical log) by €14.65 (cost per minute)*			
Direct costs (consumables), €; mean (SD)	1047 (314)	2057 (216)	** < 0.001**
*Laparoscopic: Sum of the cost of surgical consumables used in each surgery, calculated individually for each procedure* *Robotic: Sum of the cost of surgical consumables used in each surgery, calculated individually for each procedure* - *calculation of "one use" of surgical material with a limited number of uses (10–18 depending on the material)-* *Example: Total cost of Maryland bipolar forceps "Xi" / number of uses* = *one use per surgery*			
Direct costs (equipment depreciation for 1000 procedures), €; mean (SD)	140 (0)	2.000(0)	** < 0.001**
*Laparoscopic: Sum of the cost of the laparoscopic tower (€140,000) divided by 1000 surgical procedures* *Robotic: Sum of the cost of the Da Vinci Xi robotic system (€2,000,000) divided by 1000 surgical procedures* *The depreciation value of both types of equipment is 10 years, assuming the use of 100 procedures per year, estimating at least 1000 procedures during the depreciation period of both products*			
Indirect costs (hospital stay), €; mean (SD)	1153 (698)	768 (317)	**< 0.001**
*Calculated by multiplying the length of stay in days by the cost per day (€681)*			
Total cost per procedure (depreciation value, 1000 procedures), €; mean (SD)	4698 (1121)	7052 (816)	**< 0.001**
*Includes: Indirect costs (operating room)* + *Direct costs (consumables)* + *Indirect costs (hospital stay)* + *Direct costs (equipment depreciation for 1000 procedures)*			

LPS: Laparoscopy; RBT: Robotic

Table 3 shows the results of the multivariate analysis. The following variables, considered of clinical interest, were included: age, BMI, duration of surgery, duration of hospitalization and surgical complication. The duration of hospitalization was independently associated with robotic surgery (*p* < 0.001), suggesting that robotic surgery is associated with a higher probability of shorter duration of hospitalization. Economic cost could not be considered as there was a case of perfect separation between variables, specifically between the type of surgery (laparoscopy vs. robot and economic cost).

**Table 3:** Multivariate analysis of clinical variables of interest (logistic regression)

Variable	Estimate	Std. error	*z* value	*P* value
Robotic surgery (intercept)	0.8842879	1.5486606	0.571	0.568
Age	0.0113124	0.0163193	0.693	0.488
BMI (body mass index)	0.0372578	0.0282775	1.318	0.188
Surgery duration (min)	−0.0008814	0.0037453	−0.235	0.814
Hospital stay (days)	−1.9445094	0.4213475	−4.615	** <0.001**
Surgical complication (yes)	0.6337070	1.1371937	0.557	0.577

### Theoretical model based on economy of scale

Regarding the number of cost-effective procedures that can be performed, we must consider a few assumptions. On one hand, we considered that the ideal average length of stay for a minimally invasive procedure is one day. To develop an economy of scale model we will use a ratio in which the numerator represents the total number of surgeries, and the denominator represent the total number of days of admission for that number of surgeries (calculated for each group). On the other hand, this ratio limits the number of surgeries to be performed in a period without exceeding the average stay of one day. The value 1 would be a perfect ratio where all surgeries would be admitted for 1 day. The closer it is to zero, the less procedures can be performed to prevent from increasing the mean duration of the hospital stay in more than one day of hospitalization. In our sample, laparoscopic and robotic surgery resulted in a ratio of 0.59 and 0.88, respectively. On the other hand, we considered 300 as the maximum number of surgical sessions per year (considering holidays and nonworking days) in the best-case scenario [14, 15]. We develop three theoretical assumptions (Table 4) considering an incremental number of procedures per surgical session.

**Table 4:** Theoretical economic model based on revenue ratio adjusted to not exceed one day of hospital stay

Total intervention cost per Proc	LPS ratio (0.59)	RBT ratio (0.88)	*P* value	RBT cost overrun	Increase of #Proc by RTB use
Model#1 Ratio: 1 surgery per surgical session in 10 years					
- LPS: 1770 Proc—RBT: 2640 Proc	4637€	5810€	** <0.001**	+ 1,173 € Pp	+ 87 Proc /year + 870 Proc /10 years
Model#2 Ratio: 2 surgeries per surgical session in 10 years					
- LPS: 3540 Proc - RBT: 5280 Proc	4596€	5,431€	** <0.001**	+ 835 € Pp	+ 174 Proc /year + 1740 Proc /10 years
Model#3 Ratio: 3 surgeries per surgical session in 10 years					
- LPS: 5310 Proc - RBT: 7920 Proc	4583€	5305€	** <0.001**	+ 722 € Pp	+ 261 Proc /year + 2610 Proc /10 years

LPS: Laparoscopy; RBT: Robot; Pp: Per surgical procedure; Proc: Surgical procedure

In Model#1 Ratio (Table 4), we consider scheduling 1 surgical procedure per surgical session. The maximum number of procedures was adjusted to not exceeding the average of 1 day of admission by multiplying by the ratio of each group. In the laparoscopy group resulted in 177 procedures per year (300 × 0.59) that can be performed adjusted to an average of 1 day of hospital admission. Therefore, up to 1770 patients could be operated in the amortization period (10 years). Applying the same calculation for robotic surgery (300 × 0.88), we could operate on 2640 patients in that period. If we adjust the direct costs of the equipment to this number of interventions, the average cost of laparoscopic and robotic surgery for a surgical procedure per surgical session, adjusted to an average of 1 day of hospitalization, would be €4637 and €5810, respectively (*p* < 0.001), being the robotic procedure 20.2% more expensive (+ 1173€). The Model#2 Ratio (Table 4), considered planning 2 surgical procedures per session, adjusting the total number of operated patients by the ratio over the 10-year amortization period. Thus, the average cost of laparoscopic and robotic surgery would be €4596 vs. €5431, respectively (*p* < 0.001), being the robotic procedure €835 more expensive than laparoscopy (15.4%). Finally, the Model#3 Ratio (Table 4), considered planning 3 surgical procedures per session, adjusting the total number of operated patients by the ratio over the 10-year amortization period. The cost of laparoscopic and robotic surgery would be €4583 vs. €5305, respectively (*p* < 0.001), being the robotic procedure €722 more expensive (13.6%).

Cost minimization analysis: theoretical simulation model of robotic surgery costs per procedure by applying discount on robotic equipment.

**Fig. 2:**
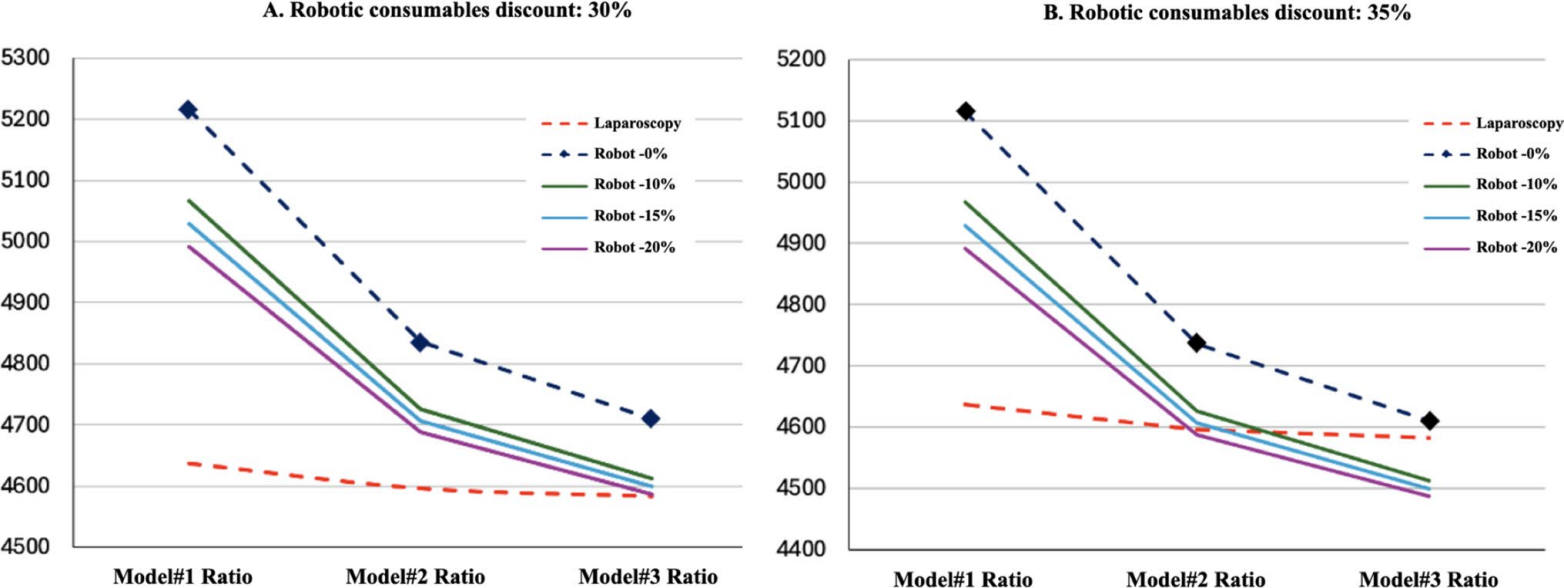
Theoretical simulation model of robotic surgery costs per procedure by applying discount on robotic equipment

Finally, a model based on the reduction of robotic surgery costs from a theoretical point of view is developed in search of a scenario that equals the costs between laparoscopic and robotic approaches. The cost per procedure could be reduced by applying discounts on the fixed cost of purchasing robotic equipment and/or on the cost of consumables used per procedure combined with the theoretical ratio admission adjusted model (Table 4). Figure 2 shows the evolution of the cost per procedure in different scenarios of reducing the cost of robotic consumables by 30% (Fig. 2A) and 35% (Fig. 2B). The horizontal axis shows the ratio-models analyzed, while the vertical axis shows the cost per procedure. Laparoscopy (dashed red line) maintains the lowest and most stable cost in all models. Nondiscounted robotic surgery (dashed blue line with diamond-shaped markers) has a high initial cost that decreases across the models. With the application of discounts on equipment acquisition of 10%, 15% and 20%, a progressive reduction in cost per procedure is observed. In model 1, robotic surgery is still more expensive than laparoscopy, but in models 2 and 3, the values converge, becoming equal to or even less expensive than laparoscopy.

## Discussion

### Summary of main results

This study demonstrates that robotic surgery for early-stage endometrial cancer incurs higher costs when compared with laparoscopic surgery without offering significant clinical advantages. Although the robotic approach results in a shorter hospital stay, it does not improve surgical time or complication rates. However, under certain conditions—such as increasing the number of robotic surgeries per session and applying discounts on equipment and consumables—robotic surgery can become economically competitive. A minimum of 2–3 robotic surgeries per session is recommended to reduce costs. However, it is also necessary to apply discounts in the acquisition of robotic equipment (10–15%) and consumables (30–35%). An alternative to applying this discount on consumables would be to extend the lifespan of robotic instruments by increasing its number of uses.

### Results in the context of published literature

The clinical outcomes observed in this study align with previous European findings. In a comparative analysis, the average operative time was slightly shorter for robotic surgery (210 min) compared to laparoscopy (214 min). Robotic surgery had fewer intraoperative complications (2.1% vs. 6.2%) and a shorter average hospital stay (3.2 days vs. 4.7 days), although postoperative complications were slightly higher for robotic surgery (26% vs. 23%). Transfusion rates were similar across both groups. Cost analysis showed robotic surgery to be more expensive, with an average total cost of €4462 compared to €3746 for laparoscopy—an increase of €716 or 16%. Given that laparoscopy is the current standard for treating endometrial cancer, robotic surgery must either demonstrate superior clinical results or prove more economical. The latter could be achieved under specific conditions such as increased procedure volume, extended instrument use, and modest equipment discounts [6–12].

This study provides evidence that may serve as a supporting argument in negotiations for the acquisition and implementation of new medical technologies. In Spain, where 96.6% of the population relies on the National Health System [16], public healthcare spending reached €99.3 billion in 2023, equivalent to 7.4% of GDP. Much of this increase is attributed to the development and adoption of new technologies. However, it is not merely the technologies themselves that raise costs, but how they are integrated and utilized within the healthcare system. Medical technologies can be classified into three categories: those with broad application and strong evidence, those used for specific populations regardless of cost-effectiveness, and those with limited benefit or insufficient scientific support. Robotic surgery currently straddles the second and third categories depending on its clinical use. Therefore, its continued implementation must be carefully managed to ensure both clinical value and financial sustainability [17].

### Strengths and weaknesses

A major strength of this study is its single-center design, which ensures consistent surgical standards. Limitations include its retrospective approach and reliance on a theoretical economic model, which may vary in relevance across different health systems.

### Implications for practice and future research

Procurement decisions should consider not only the economic cost but also the cost-effectiveness of adopting new technologies. Future research should aim to validate these economic models in real-world settings to support evidence-based decision-making.

## Conclusions

Robotic surgery for endometrial cancer is linked to shorter hospital stays. When performed at a rate of two to three surgeries per day, with a 10% equipment discount and 35% savings on consumables, robotic surgery can match or even undercut the cost of laparoscopy.

## Data availability statement

Data will be available for future meta-analysis or for verification of the results upon reasonable and formal request.

## Supplementary Information

Below is the link to the electronic supplementary material.

Supplementary file1 (PDF 149 KB)
