# Supplementary material for: Endo-cost: efficient economic model of adopting robotic versus laparoscopic gynecological surgery for endometrial cancer
Source: J Robot Surg. 2025 Nov 3;19(1):744. doi: 10.1007/s11701-025-02706-6 (PMC12583328; doi:10.1007/s11701-025-02706-6)
Supplement: Supplementary file 1 — Supplementary file1 (PDF 149 KB) [file 11701_2025_2706_MOESM1_ESM.pdf]

OPINION OF THE RESEARCH ETHICS COMMITTEE WITH MEDICINAL PRODUCTS

JUAN EDUARDO MEGIAS VERICAT, Secretary of the Technical Secretariat of the Research Ethics Committee with Medicinal Products (CEIM) - **UNIVERSITY AND POLYTECHNIC HOSPITAL LA FE**,

CERTIFIES

That this Committee evaluated, in its session dated **September 4, 2024**, meeting No. 574, the sponsor's proposal to carry out the observational study involving medical devices:

- **Protocol Code**: END-COST
- **Title**: *"Economic analysis of the adoption of medical technology: Efficiency of robotic gynecologic oncology surgery compared to laparoscopic surgery."*
- **Sponsor**: VÍCTOR LAGO LEAL
- **Principal Investigator / Coordinator**: VÍCTOR LAGO LEAL

Documents Reviewed:

- Protocol – July 30, 2024
- Request for exemption from informed consent – July 30, 2024
- Financial report – July 30, 2024

The committee evaluated the proposed economic compensations and their possible interference with ethical principles.

The study complies with ethical standards outlined in current legislation, the Declaration of Helsinki by the World Medical Association on ethical principles for medical research involving human subjects and its subsequent revisions, as well as applicable regulations according to the study characteristics.

Favorable Opinion Issued for the conduct of the study.

The CEIM – UNIVERSITY AND POLYTECHNIC HOSPITAL LA FE, both in its composition and procedures, complies with Good Clinical Practice guidelines (CPMP/ICH/135/95) and current regulations governing its operation. If any member of the CEIM was involved in the study or had a conflict of interest, they did not participate in the evaluation or approval process.

Signed in Valencia, on September 4, 2024

Signed: JUAN EDUARDO MEGIAS VERICAT

Secretary of the Technical Secretariat of CEIM

CSV: 96ZS6CY1:5SGDE7UQ:MMVVYIRI

Validation URL: https://www.tramita.gva.es/csv-front/index.faces?cadena=96ZS6CY1:5SGDE7UQ:MMVVYIRI

ANNEX I – COMPOSITION OF THE CEIM

- **President**: ÓSCAR DÍAZ CAMBRONERO – Specialist in Anesthesiology and Resuscitation, Section Head
- **Vice President**: PAULA RAMÍREZ GALLEYMORE – Specialist in Intensive Care Medicine
- **Secretary**: JUAN EDUARDO MEGIAS VERICAT – Specialist Pharmacist in Hospital Pharmacy

Members (Vocales):

- MARÍA VICTORIA PARICIO GÓMEZ – Nursing Supervisor, Hematology and Stem Cell Transplant Unit
- SERAFÍN RODRÍGUEZ CAPELLÁN – Lawyer, Administrative Function Technician
- VICENTE INGLADA ALCAIDE – Lay Member, Patient Representative
- MIGUEL ÁNGEL CANO TORRES – Lawyer, Administrative Function Technician
- LUIS VICENTE MARTÍNEZ DOLZ – Specialist in Cardiology, Service Head
- MATTEO FRASSON – Specialist in General and Digestive Surgery
- ANTONIO ORDUÑA GALÁN – Systems Engineer, Head of Security and Information Systems Quality
- ANA PEIRÓ PEIRÓ – Specialist in Clinical Pharmacology
- MARÍA TORDERA BAVIERA – Specialist Pharmacist in Hospital Pharmacy
- GUIDO MAZZINARI – Specialist in Anesthesiology
- PABLO SOPENA NOVALES – Specialist in Nuclear Medicine
- JOSÉ MARÍA CANELLES GAMIR – Primary Care Pharmacist
- AMPARO SOLER DIEGO – Medical Coordinator, Health Centers Miguel Servet and Azucena
- JUAN PABLO REIG MEZQUIDA – Specialist in Pulmonology
- MARTA AGUAR CARRASCOSA – Specialist in Pediatrics, Neonatology Unit
- DAVID MARTÍNEZ CUADRÓN – Specialist in Hematology and Hemotherapy, Researcher
- FRANCISCO CARLOS PÉREZ MIRALLES – Specialist in Neurology

CEIm-F-PE-01-11 v01

DICTAMEN DEL COMITÉ DE ÉTICA DE LA INVESTIGACIÓN CON MEDICAMENTOS

JUAN EDUARDO MEGIAS VERICAT, titular de la Secretaría Técnica del Comité de Ética de la Investigación con medicamentos del **CEIM - HOSPITAL UNIVERSITARIO Y POLITÉCNICO LA FE,**

CERTIFICA

Que este Comité ha evaluado, en su sesión de fecha 04/09/2024, con acta nº 574, la propuesta del promotor para que se realice el estudio observacional con productos sanitarios:

Código de protocolo: **END-COST**
Título: **"Análisis económico de la adopción de tecnología médica: Eficiencia de la cirugía oncológica ginecológica robótica comparada con cirugía laparoscópica."**
Promotor: **VÍCTOR LAGO LEAL**
Investigador principal/ coordinador: **VÍCTOR LAGO LEAL**

Documento	Versión - Fecha
Protocolo	30 de Julio de 2024
Solicitud de exención del consentimiento informado	30 de Julio de 2024
Memoria Económica	30-07-2024

Que se han evaluado las compensaciones económicas previstas y su posible interferencia con el respeto a los postulados éticos.

Que se cumplen los preceptos éticos formulados en la legislación vigente y la Declaración de Helsinki de la Asociación Médica mundial sobre principios éticos para las investigaciones médicas en seres humanos y en sus posteriores revisiones, así como aquellos exigidos por la normativa aplicable en función de las características del estudio.

Y emite un **DICTAMEN FAVORABLE** para la realización de dicho estudio.

Que el CEIM - HOSPITAL UNIVERSITARIO Y POLITÉCNICO LA FE tanto en su composición como en sus procedimientos, cumple con las normas de BPC (CPMP/ICH/135/95) y con la legislación vigente que regula su funcionamiento, y que la composición del CEIM - HOSPITAL UNIVERSITARIO Y POLITÉCNICO LA FE es la indicada en el Anexo I, teniendo en cuenta que en el caso de que algún miembro participe en el ensayo o declare algún conflicto de interés no habrá participado en la evaluación ni en el dictamen de la solicitud de autorización del estudio clínico.

Lo que firmo en Valencia, a 04/09/2024

Firmat per Juan Eduardo Megias Vericat, el 05/09/2024 11:41:10

GENERALITAT VALENCIANA

Fdo.: JUAN EDUARDO MEGIAS VERICAT
Titular de la secretaría técnica del CEIm

CSV:96ZS6CY1:5SGDE7UQ:MMVVYIRI URL de validació:https://www.tramita.gva.es/csv-front/index.faces?cadena=96ZS6CY1:5SGDE7UQ:MMVVYIRI

ANEXO I
COMPOSICIÓN DEL CEIm

Presidente:
ÓSCAR DÍAZ CAMBRONERO - Facultativo especialista en Anestesiología y Reanimación. Jefe Sección Anestesiología y Reanimación

Vicepresidente:
PAULA RAMÍREZ GALLEYMORE - Facultativo especialista en Medicina Intensiva. Médico adjunto del Servicio de Medicina Intensiva

Secretario:
JUAN EDUARDO MEGIAS VERICAT - Farmacéutico especialista en Farmacia Hospitalaria. Farmacéutico adjunto Unidad de Ensayos Clínicos

Vocales:
MARÍA VICTORIA PARICIO GÓMEZ - Diplomada Enfermería. Supervisora del Servicio de Hematología y Trasplante de Progenitores Hemáticos
SERAFÍN RODRÍGUEZ CAPELLÁN - Licenciado en Derecho. Técnico de Función Administrativa adscrito a la Dirección de Investigación
VICENTE INGLADA ALCAIDE - Miembro Lego, representante de los intereses de los pacientes
MIGUEL ÁNGEL CANO TORRES - Licenciado en Derecho. Técnico de Función Administrativa adscrito a la Dirección de Investigación
LUIS VICENTE MARTÍNEZ DOLZ - Facultativo especialista en Cardiología. Jefe de Servicio de Cardiología
MATTEO FRASSON - Facultativo especialista en Cirugía. Médico adjunto del Servicio de Cirugía General y Digestiva
ANTONIO ORDUÑA GALÁN - Ing. de aplicaciones y sistemas. Responsable Área de Seguridad y Calidad de Sistemas de Información
ANA PEIRÓ PEIRÓ - Fac. especialista en Farmacología Clínica. Médica Adjunta del Servicio Farmacología Clínica. DSA-HG.
MARÍA TORDERA BAVIERA - Farmacéutica especialista en Farmacia Hospitalaria. Farmacéutica adjunta del Servicio de Farmacia
GUIDO MAZZINARI - Facultativo especialista en Anestesiología y Reanimación. Médico adjunto del Servicio de Anestesia.
PABLO SOPENA NOVALES - Facultativo especialista en Medicina Nuclear. Médico adjunto del Área de Imagen Médica
JOSÉ MARÍA CANELLES GAMIR - Farmacéutico de Atención Primaria del Departamento de Salud Valencia La Fe
AMPARO SOLER DIEGO - Coordinadora médica del CS Miguel Servet y CS Azucena del Departamento de Salud Valencia - La Fe
JUAN PABLO REIG MEZQUIDA - Facultativo especialista en Neumología. Médico adjunto de la Unidad de Trasplante Pulmonar y FQ
MARTA AGUAR CARRASCOSA - Facultativo especialista en Pediatría. Médica adjunta del servicio de Neonatología.
DAVID MARTÍNEZ CUADRÓN - Facultativo especialista en Hematología y Hemoterapia. Investigador IIS La Fe
FRANCISCO CARLOS PÉREZ MIRALLES - Facultativo especialista en Neurología. Médico adjunto del servicio de Neurología

CSV:96ZS6CY1:5SGDE7UQ:MMVVYIRI **URL de validació:**https://www.tramita.gva.es/csv-front/index.faces?cadena=96ZS6CY1:5SGDE7UQ:MMVVYIRI
